# Is Age Just a Number? Influence of Caregiver Age on Injuries of Children Raised by Grandparents

**DOI:** 10.1093/geroni/igab046.3037

**Published:** 2021-12-17

**Authors:** Acacia Lopez, Rachel Scott, Danielle Nadorff

**Affiliations:** 1 Mississippi State University, Starkvile, Mississippi, United States; 2 Mississippi State University, Starkville, Mississippi, United States; 3 Mississippi State University, Mississippi State, Mississippi, United States

## Abstract

Unintentional injuries are the most common cause of death for children in the United States. One factor linked to their occurrence is parenting style (i.e., a collection of factors such as physical health, mental health, and possible cohort differences in parenting role expectations). Differences in parenting behaviors may be evident in grandparents caring for their grandchildren, due to cohort differences and age-related declines in cognitive and physiological processes. This may impact their abilities to monitor, supervise, and respond to children. Further, Hayslip & Kaminski report custodial grandparents are less likely than parents to understand and respond to the psychological and emotional needs of children but are more likely to enforce discipline. This study sought to explore the ways in which parenting styles are associated with unintentional injury behaviors in children (via caregiver age) for grandchildren raised by grandparents. Participants were grandparents raising their grandchildren, recruited via Qualtrics Panel Service (N = 323). Conditional process analyses were conducted using Model 1 of SPSS PROCESSv3.5. Age moderated the relation between consistency of discipline and child unintentional injury (F (1, 231) = 12.67, p <.001) as well as level of supervision and child unintentional injury (F (1,146) = 6.23, p = .01). Age did not moderate the relation between positive parenting and unintentional injuries. These results imply that children being raised by older grandparents were especially at risk for increased injuries when their grandparents used less consistent discipline or lower rates of supervision. Pathways are suggested for age-specific psychoeducation interventions for custodial grandparents.

